# Effects of inspiratory muscle training for 28 days on the voice of women without vocal complaints

**DOI:** 10.1590/2317-1782/e20250109en

**Published:** 2026-03-02

**Authors:** Julia Batistella, Alessandra Thais Beraldo, Perla do Nascimento Martins, Ana Paula Dassie-Leite, Eliane Cristina Pereira

**Affiliations:** 1 Departamento de Fonoaudiologia, Universidade Estadual do Centro-Oeste – UNICENTRO, Irati (PR), Brasil.

**Keywords:** Breathing Exercises, Voice, Vocal Quality, Breathing, Voice Training

## Abstract

**Purpose:**

To analyze the vocal effects of inspiratory muscle training for 28 days in women without vocal complaints.

**Methods:**

The study included 22 women with no vocal complaints who underwent inspiratory training with the Respiron® Classic Breathing Trainer and Stimulator. The training consisted of 2 sets per day of 30 repetitions for 28 consecutive days. Vocal samples were collected pre and post training and compared regarding auditory-perceptual evaluation (APE), acoustic analysis, aerodynamic and diadochokinesis measurements, and vocal self-assessment. The data was statistically analyzed using dependent tests.

**Results:**

No differences were found in the APE of the sustained vowel /a/ and number counting. Differences were found in the acoustic analysis, with a decrease in the median Shimmer % and an increase in HNR. There was an increase in the average maximum phonation times of the vowel /i/, of the fricatives /s/ and /z/, and counting numbers. There was also an increase in the average number of syllable repetitions in the diadochokinesis of the syllables /ka/ and /pataka/ and the vowel /a/. Increase in the means of the physical V-RQOL self-assessment protocol and total V-RQOL, and decrease in VHI-10; VoiSS limitation, VoiSS physical, and total VoiSS.

**Conclusion:**

It can thus be inferred that the inspiratory training resulted in enhanced phonatory efficiency and augmented aerodynamic control, thereby conferring an improvement in vocal quality. It is therefore recommended that subjects who do not present vocal complaints and who wish to enhance their phonatory efficiency engage in inspiratory muscle training.

## INTRODUCTION

Voice production depends on adequate air pressure, which is essential for the vocal folds to close and vibrate efficiently^(1-3)^. In this sense, respiratory exercisers and stimulators have been used in vocal clinics with the aim of improving phonatory efficiency, that is, the coordination between the respiratory, glottal, and resonant subsystems, which results in voice production with minimal effort and maximum use of respiratory energy^1-,4^, although there is a scarcity of studies in the literature that prove their effects.

Respiratory stimulators are often used by physiotherapists to build muscle strength, both in the inspiratory and expiratory muscles. Currently, these devices are commonly used in post-thoracic surgery recovery, as well as being indicated for increasing oxidative capacity, reducing fatigue, and improving the neuromuscular system’s performance^(4-6)^.

The Respiron® Classic Breathing Trainer and Stimulator is a device that stimulates breathing through muscular effort. These sorts of devices encourage deep inspiration, with maximum expansion of the lungs, thus stimulating muscular activity, mainly in the diaphragm and other intercostal muscles responsible for respiratory movement. Since these are muscles, they can be trained to increase strength and resistance to fatigue^(7,8)^.

The Respiron® Classic Breathing Trainer and Stimulator is easy and painless to use, using a set of weights for the inspiratory muscles. It also provides visual stimulation (raising of the spheres) that relates to the level of difficulty of the exercise performed. This model offers exercises that require moderate effort, making it ideal for sedentary, obese, and elderly people who are just starting respiratory exercises. The benefits of regular use include reduced fatigue and shortness of breath, increased willingness to engage in physical activity, improved quality of life, and physical performance^(7)^. There is a high possibility of improvement within a period of three months^(9)^, but the beginning of improvement can already be observed after the second week of use^(8)^.

Two studies investigated the immediate effect of inspiratory exercise with Respiron® Classic on the voices of women with no vocal complaints. One study involving 22 vocally healthy women found that the device is safe and that its immediate effect promoted a reduction in short-term aperiodicity acoustic measures related to frequency and intensity and an increase in maximum expiratory volume^(10)^. The second study, conducted with 25 women without vocal complaints, concluded that there is an increase in the aerodynamic measurement of TMF /s/. This result demonstrates the improvement in aerodynamic control as an immediate effect of inspiratory exercise^(11)^.

Another study using the same equipment showed that using it for four weeks increased expiratory capacity and improved laryngeal quality and efficiency. It was possible to observe an increase in PEF (peak expiratory flow) and FEV (forced expiratory volume), but without reaching the normal values expected for adults. Furthermore, the Maximum Phonation Time (MPT) values of the vowel /a/ and the consonants /s/ and /z/ increased^(12)^.

Another study assessed Maximal Inspiratory Pressure (MIP), Maximal Expiratory Pressure (MEP), and peak expiratory flow in 28 institutionalized elderly people, who underwent training using expansive manual techniques and inspiratory training with the breathing stimulator Respiron® Classic. Training was carried out three times a week for six consecutive weeks for 15 minutes, with one minute of rest every four minutes. In the aforementioned research, Respiron® Classic used alone did not change respiratory muscle strength in the elderly, however, when associated with other techniques, it contributed to an increase in respiratory muscle strength^(11)^.

Inspiratory training using breathing trainers and stimulators is currently widespread in speech therapy and is frequently used in speech therapy clinical practice. Due to the scarcity of literature elucidating its effects on multidimensional voice assessments, it is necessary to carry out studies that seek evidence on its applicability and benefits in the vocal clinic.

It is believed that breathing training aimed at improving phonatory efficiency is of great value both for people with voice disorders and for vocally healthy people who wish to improve their vocal performance in terms of quality, resonance, intensity/projection, and pneumo-phono-articulatory coordination. This is because it is believed that the increase in subglottic pressure contributes to an improvement in glottal coaptation and consequently in voice quality, an aspect that can improve diadochokinesis, which is widely used to assess the neuromotor control of the vocal folds^(13)^.

Given this, the present study aimed to compare the auditory-perceptual evaluation, acoustic measurements, aerodynamic measurements, diadochokinesis, and vocal self-assessment before and after muscle training with the Respiron® Classic Breathing Trainer and Stimulator, for 28 days in the voice of women without vocal complaints.

## METHODS

### This is a pre- and post-test intervention study.

The study was approved by the Human Research Review Board under number 5.594.862. All participants signed the Informed Consent Form (ICF).

Data gathering took place between November 2023 and March 2024. The sample consisted of 22 women, university students studying in health healthcare, with an average age of 20.90 years (+3.47), with no vocal complaints. Vocal assessment data was collected at a university voice laboratory.

On the day of the first data collection, the women were instructed on how to perform inspiratory muscle training with the Respiron® Classic Respiratory Exerciser and Incentivizer for 28 days. In order to understand how the training worked, it should be recorded in a specific protocol, as shown in Appendix A.

The participants performed a set together with the researcher and were able to ask any questions they had on the use and execution of the inspiratory training and were then instructed to perform the inspiratory training at home for 28 consecutive days. During this period, the researcher weekly contacted the participants to check whether the project was being carried out correctly and to clarify any doubts about the process.

The inclusion criteria were: female sex, undergraduate students, over 18 years old.

The exclusion criteria were the presence of vocal complaints, measured using the Screening Index for voice disorder (SIVD)^(14)^ protocol, which is a validated instrument for vocal screening and consists of a 12-question questionnaire, with each question answered "often" and "always" adding up to one point. A score of 5 or more is indicative of vocal alteration. Smokers were also excluded from the study, as were those who self-reported chronic airway alterations on the day of gathering the data; who had contracted COVID-19 up to one month before gathering the data or complications during or after COVID-19 contamination; who were undergoing or have undergone vocal treatment or laryngeal surgery; who self-reported hormonal, neurological, psychiatric, pulmonary diseases, hearing loss; and those who did not participate in any stage of the research.

### Execution of inspiratory muscle training

The participants performed the inspiratory muscle training, 2 sets per day, each with 30 repetitions, with the Respiron® Classic Breathing Trainer and Stimulator medium level, for 28 consecutive days.

All participants were instructed to perform the sets seated, inhaling strongly until the three spheres of the Respiron® Classic were raised and trying to hold for up to three seconds, being careful not to raise the shoulders and contract the muscles in the neck area.

The exercises began at level 0 with all participants, which corresponds to the minimum level of effort. In addition, participants recorded their progress in a protocol of their own, where they noted the date of training, how many times they performed during the day, and the degree of effort (from zero to three) performed on that day (Appendix A).

This study did not determine a specific level of effort for the participants to perform the training over the weeks. Therefore, the participants were instructed to increase the degree of effort (level) when all the spheres of the equipment were being lifted effortlessly at the end of the 30 repetitions.

To analyze the vocal samples, participants who performed inspiratory muscle training every day were taken into account; including participants who failed at most once a week. The researcher maintained contact with the participants in order to talk to them once a week about how the training was going and answer any questions they might have had.

### Data collection

Data and vocal samples were collected as follows:

1. Completion of the questionnaire on identification data, vocal complaints, history of illnesses, unhealthy and healthy habits, and the SIVD^(14)^ vocal screening protocol.
2. Completion of vocal self-assessment protocols: Voice-Related Quality of Life^(15,16)^, Voice Handicap Index 10 (VHI 10)^(17,18)^, and Voice Symptom Scale (VoiSS)^(19)^;
3. Voice recording in an acoustically treated laboratory, LABORVOZ, using the Audacity voice recording program on a desktop computer with an AMD Athlon (tm) 64 X2 Dual Core Processor 5000+, 4GB memory, 240GB SSD, Windows 7 Pro - 32-bit operating system, using a Shure SM58 unidirectional microphone, connected to an M Audio Fast Track audio interface, positioned in front of the mouth, at a distance of 5 centimeters. The recordings were made at a sampling rate of 44100 Hz, 16-bit resolution, mono channel, and in wave format. The recorded samples were:

- Sustained vowel /a/;
- Counting numbers from 1 to 20;
- Maximum phonation time for the vowels /a/, /i/, and /u/, phonemes /s/ and /z/, and counting numbers;
- Diadochokinesis (counting the number of syllable repetitions) with /pa/, /ta/, and /ka/, word /pataka/, and phoneme /a/.

After 28 days, the voices were recorded again, following steps 2, 3, and 4 for post-intervention collection.

Although the initial intake was 24 participants, two of them were excluded during the course of the study. One of them contracted the COVID-19 virus during training. The second had not mentioned at the time of the interview that she had gastroesophageal reflux and, at the beginning of the training session, the reflux intensified, which was reported to the researcher, who decided to discontinue the practice.

### Auditory-perceptual evaluation

The evaluator, a speech therapist specialized in voice, with 10 years of experience in vocal analysis, received a folder with the voices in pairs, without knowing which was the pre-moment and which was the post-moment. She evaluated the analyses using the Vocal Deviation Scale - VDS^(20)^, using vocal samples of the sustained vowel /a/ and counting numbers from 1 to 10, according to the overall degree of vocal deviation on a 100 mm visual analog scale.

The evaluator's internal agreement was analyzed using the Intraclass Correlation Coefficient (ICC) statistical test, repeating 20% of the sample, randomly, referring to the Vocal Deviation Scale. After analysis by the ICC, it was found that the pairs were in excellent internal agreement all with responses above 0.9.

### Acoustic voice analysis

The software PRAAT was used to carry out the acoustic analysis, extracting the acoustic measurements. The 3 most stable seconds of the sustained /a/ vowel utterance were considered and with this sample, the measurements of fundamental frequency,disturbance measurements (jitter and shimmer), and Harmonic to Noise Ratio (HNR) were obtained . The measure Cepstral Peak Prominence-Smoothed (CPPS) was extracted from the sustained vowel /a/ and the number counting^(21)^. The Acoustic Voice Quality Index (AVQI) multiparametric measure was also obtained, considering vowel samples /a/ and number counting (1 to 11)^(22)^.

The Acoustic Breathiness Index (ABI) measure was also extracted, analyzing vowel and counting together, as in the AVQI^(23)^.

### Aerodynamic measurements

In order to analyze the aerodynamic measurements, the Maximum Phonation Time of the sustained vowels /a/, /i/, /u/, and the voiceless and sonorous fricatives /s/ and /z/ were measured in seconds, then their ratio was obtained^(1)^. Each sample was collected once and time was extracted using the Audacity program.

### Diadochokinesis

The analysis of the vocal sample of diadochokinesis was performed considering the second medial of the syllable repetition, counting the number of repetitions in one second^(23)^.

### Vocal self-assessment

The following protocols were used to carry out the vocal self-assessment:

- Voice-Related Quality of Life (V-RQOL)^(15,16)^: Domain scores range from 0 to 100, with 0 indicating low voice-related quality of life and 100 indicating excellent voice-related quality of life. The cutoff values established for V-RQOL are 91.25 for the Total Domain, 89.60 for the Physical Domain, and 90.64 for the Socio-Emotional Domain^(16)^.

-Voice Handicap Index-10 (VHI-10)^(17-25)^: The total score is calculated through the simple sum of the answers ranging from 0 to 40 points, with 0 indicating no vocal handicap and 40 severe handicap. The cutoff value established for VHI-10 is 7.5 points^(17)^.

-Vocal Signals and Symptoms Scale (VoiSS)^(18)^: The cut-off values established for the VSS are 16 for the Total domain; 11.55 for the limitation subscale, 1.5 for the emotional subscale, and 6.5 for the physical subscale^(18)^.

### Statistical analysis

The study's dependent variables (pre- and post-training) are data from the auditory-perceptual evaluation, acoustic analysis, aerodynamic measurements, and diadochokinesis. As this is an intervention study, the independent variable is the 28-day training period, i.e., the time between the first and second vocal assessments.

The results were compared by analyzing samples from pre- and post-training. Prior to inferential analysis of the results, the Shapiro-Wilk normality test was performed to analyze the normality of the data distribution. Thus, Wilcoxon tests were used to compare variables with non-normal distribution and paired Student's t-tests were used to compare variables with normal distribution. In cases of statistically significant differences, Cohen's d test was used to analyze the effect size after the paired Student's t-test, and (r) was calculated to analyze the effect size after the Wilcoxon test.

The sample size was calculated using the Two Proportions Test based on a similar study that analyzed the effect of the Shaker Plus respiratory stimulator before and after three minutes of exercise. The fixed parameters adopted for the test were α of 5%, β of 20%, and K of 80%.

The minimum sample size calculated was 20 participants in the pre- and post-training groups.

## RESULTS

The study included 22 women with a mean age of 20.90 years (3.47). All participants were university students and the vocal samples were made up of women without vocal complaints, which were determined by the SIVD screening index with a score of less than 5^(15)^.

Table 1 shows the descriptive analysis of the levels of effort that each woman exerted during inspiratory muscle training before and after 28 days. It can be observed that most participants increased their level of effort throughout the training period (Level 0 – pre: 22/post: 4; Level 1 – pre: 0/post: 7; Level 2 – pre: 0/post: 10; Level 0 – pre: 0/post: 1).

**Table 1:** Distribution of effort levels performed in inspiratory muscle training pre and post use of the breathing trainer and stimulator for 28 days (n=22)

**MOMENT**	**EFFORT LEVEL**			
	Level 0	Level 1	Level 2	Level 3
Pre	22 (100%)	0 (0%)	0 (0%)	0 (0%)
Post	4 (18.18%)	7 (31.81%)	10 (45.45%)	1 (4.45%)

Source: The authors (2025)

Table 2 shows the values of the auditory-perceptual evaluation of the general degree of vocal deviation of the sustained vowel /a/ and the counting of numbers before and after the completion of the inspiratory training for 28 days. There were no significant differences in the comparisons.

**Table 2:** Distribution of perceptive-auditory evaluation values of the general grade of vocal deviation of the vowel /a/ and number counting pre and post inspiratory muscle training with breathing trainer and stimulator for 28 days (n=22)

**GENERAL DEGREE OF VOCAL DEVIATION OF THE VOWEL**							
		Mean	Median	Min.	Max.	Standard deviation	P
**Vowel /a/**	Pre	42.36	42.00	24.00	55.00	7.88	0.495
	Post	43.09	44.50	30.00	57.00	8.57	
**Number Count**	Pre	29.04	26.00	10.00	49.00	12.09	0.380
	Post	28.13	20.00	11.00	54.00	15.15	

Note: Wilcoxon test. Source: The authors (2025)

Table 3 shows the results for the acoustic values obtained before and after performing the exercise with a respiratory incentive device for 28 days. There was a significant difference between the results for shimmer (%) (pre: 2.58/post: 1.91) with a moderate to large negative effect size, and HNR (dB) (pre: 21.04/post: 22.46), with a moderate to large positive effect size. No differences were obtained for the other measures.

**Table 3:** Distribution of acoustic measurement values and self-assessment protocols before and after 28 days of inspiratory muscle training with a breathing exerciser and incentive device (n=22)

ACOUSTIC MEASUREMENT VALUES									
Group		Mean	Median	Min	Max.	Standard deviation	P	Size effect	
**F0 (%)** 2	Pre	214.53	227.11	111.95	247.97	31.13	0.338		
	Post	217.36	223.31	157.04	250.09	25.81			
**F0 numbers (%) ^2^**	Pre	210.81	216.05	161.61	214.41	20.39	0.262		
	Post	225.87	219.19	137.29	531.56	72.67			
**Jitter (%)** ^2^	Pre	0.414	0.407	0.189	250.09	25.81	0.485		
	Post	0.430	0.427	0.252	0.993	0.183			
**Shimmer (%)** ^2^	Pre	2.58	2.483	1.129	5.721	0.98	^*^ 0.008	-0.56	
	Post	1.915	1.693	0.572	5.094	0.99			
**HNR (%)** 1	Pre	21.04	21.30	16.060	28.567	2.84	*0.026	0.51	
	Post	22.46	22.74	16.331	29.698	3.68			
**CPPS /a/ (dB)** ^1^	Pre	14.75	14.48	12.29	17.87	1.56	0.479		
	Post	14.90	15.02	11.39	17.48	1.69			
**CPPS numbers (dB)** ^1^	Pre	9.03	8.81	7.04	11.06	1.02	0.428		
	Post	9.14	9.19	6.21	11.07	1.27			
**AVQI** ^1^	Pre	1.665	1.47	0.14	3.030	0.83	0.778		
	Post	1.698	1.68	0.080	3.45	0.91			
**ABI** ^1^	Pre	2.946	2.78	1.700	5.080	0.84	0.866		
	Post	2.921	2.835	1.340	4.220	0.76			
**VALUES ​​OF SELF-ASSESSMENT PROTOCOLS**									
	Group	Mean	Median	Min	Max	Standard deviation	p	Size effect	
	**QUALITY OF LIFE IN VOICE**								
**Physical Domain** ^2^	Pre	88.25	91.70	66.70	100	9.58	*0.00	0.88	
	Post	93.17	95.80	79.20	100	7.21			
**Social-Emotional Domain** ^2^	Pre	96.88	100	87.50	100	4.61	0.10		
	Post	98.027	100	93.80	100	2.95			
**Total** ^2^	Pre	91.81	93.75	80	100	6.46	*0.00	0.88	
	Post	95.25	97.50	87.50	100	4.75			
	**VOCAL HANDICAP INDEX-10**								
	Pre	3.00	2.00	0.00	18.00	4.05	*0.005	-0.89	
	Post	1.81	1.00	0.00	9.00	2.44			
	**VOCAL SYMPTOM SCALE**								
**Limitation** ^2^	Pre	8.04	8.00	0.00	21.00	5.60	*0.00	-0.86	
	Post	6.04	6.00	0.00	16.00	4.78			
**Emotional** ^2^	Pre	0.63	0.00	0.00	6.00	1.49	0.108		
	Post	0.45	0.00	0.00	6.00	1.40			
**Physical** ^2^	Pre	4.81	4.50	1.00	12.00	2.80	*0.007	-0.82	
	Post	3.86	3.00	0.00	10.00	2.49			
**TOTAL** ^2^	Pre	13.68	12.00	2.00	28.00	7.54	*0.000	-0.88	
	Post	10.72	8.50	1.00	25.00	6.65			

* Significant values

Note Acoustic Measures: ^1^Student's t-test for dependent variables with d Cohen

2 Wilcoxon test and effect size with effect r.

Source: The authors (2025)

A comparison of vocal self-assessment was also performed using the Voice Quality of Life (VQOL)16, Vocal Handicap Index-10 (VHI-10)17, and Vocal Signs and Symptoms Scale (VSSS)18 protocols, before and after 28 days of inspiratory training (Table 3). Significant differences were observed in the median values of the QOL protocols for the physical domain (pre 91.70 and post 95.80, p-value: 0.00 and effect size 0.88) and total domain (pre 93.75 and post 97.50, p-value: 0.00 and effect size 0.88), in the IDV-10 protocol (pre: 2 and post: 1, p-value: 0.005, effect size -0.89) and in the ESV limitation scale (pre: 8 and post: 6, p-value: 0.00, effect size -0.86) physical (pre: 4.5 and post: 3, p-value: 0.007, effect size -0.82) and total (pre: 12 and post: 8.5, p-value: 0.00, effect size -0.88). All of the above effect sizes (r) are considered large, indicating substantial impacts on the outcomes analyzed.

Table 4 shows the aerodynamic measurements for maximum pre- and post-phonation times. Significant differences can be observed, with increased values in the post-measurements for the vowel /i/ (pre: 13.86/post: 15.90), the fricatives /s/ (pre: 13.36/post: 16.04) and /z/ (pre: 13.54/post: 16.18), and number counting (pre: 17.68/post: 19.50). In all cases of differences, there was a moderate to large positive effect size.

**Table 4:** Distribution of maximum phonation times before and after inspiratory muscle training with a breathing trainer and stimulator for 28 days (n=22)

MAXIMUM PHONATION TIMES							
		Mean	Median	Min	Max.	Standard deviation	P
**MPT /a/** 2	Pre	14.22	13.00	9.00	29.00	4.81	0.159
	Post	15.09	14.50	8.00	25.00	3.97	
**MPT /i/** 1	Pre	13.86	14.00	8.00	23.00	3.31	^*^ 0.045
	Post	15.90	15.00	9.00	28.00	4.50	
**MPT /u/** ^1^	Pre	13.63	14.00	8.00	22.00	3.23	0.129
	Post	14.90	14.00	10.00	23.00	3.13	
**MPT /s/** ^1^	Pre	13.36	14.50	5.00	24.00	4.42	*0.034
	Post	16.04	16.00	6.00	27.00	5.68	
**MPT /z/** ^2^	Pre	13.54	13.00	6.00	27.00	4.76	*0.014
	Post	16.18	15.00	9.00	34.00	5.59	
**MPT number counting^1^**	Pre	17.68	17.50	11.00	29.00	4.49	*0.016
	Post	19.5	19.50	11.00	37.00	5.73	
**s/z ratio^1^**	Pre	1.01	0.96	0.60	1.63	0.28	0.79
	Post	0.995	1.05	0.62	1.35	0.22	

* Significant values

1 Student's t-test for dependent variables with d Cohen

2 Wilcoxon test and effect size with effect r.

Source: The authors (2025)

Table 5 shows the values referring to the number of repetitions of the diadochokinesis test of the syllables /pa/, /ta/, /ka/ /pataka/ and the vowel /a/ before and after. Significant differences were observed, with an increase in the number of syllables produced in the post-test in the measurements of /ka/ (pre: 5.90/post: 6.68), /a/ (pre: 4.63/post: 5.40) and /pataka/ (pre: 7.72/post: 8.45). The statistical differences had a moderate to large effect size.

**Table 5:** Distribution of diadochokinesis values pre and post inspiratory muscle training with breathing trainer and stimulator for 28 days (n=22)

DIADOCHOKINESIS VALUES								Size effect
		Mean	Median	Min	Max.	Standard deviation	p	
**PA**	Pre	6.36	7.00	4.00	7.00	0.84	0.806	
	Post	6.50	7.00	5.00	8.00	1.144		
**TA**	Pre	6.40	6.00	4.00	8.00	1.05	0.147	
	Post	6.81	7.00	5.00	9.00	1.18		
**KA**	Pre	5.90	6.00	4.00	8.00	1.01	*0.012	0.58
	Post	6.68	6.50	5.00	9.00	1.12		
**A**	Pre	4.63	5.00	3.00	6.00	0.78	*0.005	0.67
	Post	5.40	5.00	4.00	7.00	0.85		
**PATAKA**	Pre	7.72	8.00	6.00	10.00	0.98	*0.009	0.65
	Post	8.45	8.50	7.00	10.00	0.96		

* Significant values

Wilcoxon test and effect size with effect r.

Source: The authors (2025)

## DISCUSSION

Inspiratory training comprises a series of exercises designed to enhance the coordination of the muscle groups essential for respiration. It is understood that these muscles must function in concert to provide the requisite amount of air for the efficient production of vocal sounds. Therefore, inspiratory training is relevant in clinical speech therapy practice, as it influences transglottic airflow and subglottic pressure, favoring vocal production and contributing to improved vocal quality^(3,11)^.

The study participants commenced the respiratory training at a level of effort that was deemed to be zero, and no effort level was set for the conclusion of the training. As evidenced in Table 1, the majority of participants completed the training at effort level two, with only one individual advancing to the final level. A study^(5)^ adapted a manovacuometer to the Respiron® Classic breathing trainer and stimulator, with the aim of investigating the resistance generated at the different levels of the Respiron® Classic equipment in young individuals, using the maximum inspiratory pressure. The study concluded that the resistance generated by the equipment is mild to moderate for levels 1, 2, and 3. Additionally, the variations in effort levels were found to significantly impact the final measurements of maximum inspiratory pressure.

No differences were observed in the auditory-perceptual evaluation of the general degree of vocal deviation using the Vocal Deviation Scale (VDS) between the pre-and post-training periods. The data indicate that the device did not result in perceptual-auditory changes in vocal quality when used for the designated time period. Given that the training program exclusively addressed respiratory aspects, without incorporating laryngeal elements, it was hypothesized that there would be minimal to no significant alterations in the APE, particularly in individuals without symptoms and without notable changes in the deviation scale. Research involving the respiratory training of people with vocal disorders may yield different results. This is because the laryngeal changes that occur as a result of such training are more significant and thus have a greater impact on changes in vocal quality that are more readily perceived from a perceptual-auditory standpoint. In light of these findings, further investigation into this population is recommended.

In a study examining the immediate effects of using the Respiron® Classic Breathing Trainer and Stimulator, no changes in vocal quality were observed through auditory-perceptual judgment^(10)^. It is a common outcome of research to find no differences in auditory-perceptual judgment and differences in acoustic measurements. This is because researchers attempt to relate acoustic measurements with vocal quality perceived auditorily. However, changes in isolated acoustic parameters do not usually perfectly reflect what the clinician hears^(26)^.

Another study investigating the immediate effects of using Respiron® Classic in vocally healthy women11 showed that vocal quality improved in 36.4% of participants after using an inspiratory device, while 50.0% did not experience any noticeable changes. The authors mention that because these were non-dysphonic women, changes in vocal quality would be more difficult to perceive due to the homeostasis of the phonatory system, and suggest further studies with dysphonic subjects.

Acoustic analysis represents the most frequently utilized procedure in the domain of voice for pre- and post-intervention monitoring, offering distinctive estimates regarding alterations in the vocal production process^(27)^. It is worth highlighting that the women in this research did not present vocal complaints, and it is expected that the measurement values are within or very close to normal standards in the analysis of the pre-use of the breathing trainer and stimulator. Even so, there were changes in the parameters.

The present study demonstrated that the training resulted in notable alterations in the measurements shimmer % and dB (p=0.008), with a median value of 2.483 in the pre-test and 1.693 in the post-test. These findings indicate a reduction in the measurement value, which is within the expected range for women, as defined by the standard deviation of 1.393 to 4.861^(28)^. The shimmer measure is utilized to assess the stability of the vocal folds. It is known to be susceptible to alterations when there is a reduction in glottal resistance, the presence of mass lesions in the vocal folds, and an increase in noise during emission^(29)^. This finding corroborates the results of a study^(10)^ in which there was a significant reduction in jitter (p<0.001), shimmer (p=0.011), and PPQ (p=0.001) values, indicating an improvement in voice stability and regularity.

The HNR% (p=0.026) demonstrated a notable increase in the mean value, indicating a rise in the proportion of harmonics relative to noise. The pre-and post-treatment averages were 21.04 and 22.46, respectively, both falling within the anticipated range for the measured values, which ranged from 12.64 to 26.51^(28)^. The Harmonic to Noise (HNR %) is a measure of the efficiency of the phonation process. It is calculated by dividing the energy in the fundamental frequency by the energy in the noise frequency. A higher HNR^(30)^ ratio indicates a more efficient and intact vibratory cycle. The data presented here differ from those of another study^(10)^, which did not observe any acoustic changes following the use of Respiron®. However, the aforementioned research was based on an analysis of the immediate effect and did not extend over a period of 28 days, as was the case in the present study.

A study that compared the immediate effect of inspiratory exercise with a Respiron® Classic Breathing Trainer and Stimulator found no significant changes in acoustic measurements^(11)^.

The present research identified that the use of the device Respiron® Classic helped to reduce the shimmer measures and increase the HNR. These changes were observed to occur within the normality standard, yet they were nonetheless statistically significant. A reduction in shimmer measurements and an increase in HNR indicate an improvement in the acoustic quality of the recordings. It can thus be seen that the use of the trainer and respiratory stimulator contributes to an increase in speech efficiency since both measures are related to the amount of air necessary for phonation.

Modifications to the breathing pattern have the potential to enhance diaphragmatic capacity, thereby augmenting respiratory lung function and air storage capacity. The utilization of a respiratory stimulator has been demonstrated to exert an influence on respiratory dynamics, thereby facilitating adequate aerodynamic flow and consequently enhancing vocal control and prolonging emission time^(3,12)^. The present study revealed significant differences in the maximum phonation times of the vowels /i/, the consonants /s/ and /z/, and number counting.

A study on the immediate effect of using the e Respiron® Classic Breathing Trainer and Stimulator also observed an increase in the mean phonetic value of the phoneme /s/. The authors proposed that an increase in this measure indicates enhanced aerodynamic control and augmented lung capacity^(11)^. This present study demonstrated the efficacy of inspiratory training in modifying aerodynamic measurements during a single performance. Similar outcomes were identified in a study involving five vocally healthy individuals^(12)^, wherein an augmentation in MPT was discerned in the final production averages of vowels and fricatives. This phenomenon suggests that the utilization of respiratory stimulators may facilitate enhanced aerodynamic control. Furthermore, the research demonstrated that the observed improvement was maintained for a period of five weeks following the cessation of stimulator use. These findings corroborate a study^(10)^ that showed improvement in aerodynamic measures, with a significant increase in maximum expiratory volume (p<0.001), suggesting an improvement in respiratory capacity.

The aerodynamic measurement of the MPT is directly correlated with the aerodynamic flow of breathing and phonation, as well as the myoelastic forces exerted by the larynx^(11,12)^. In light of the existing literature and the findings of the present study, it can be posited that the utilization of the Respiron® Classic Breathing Trainer and Stimulator facilitates augmented aerodynamic control and myoelastic forces of the larynx.

The diadochokinesis test is a common assessment tool in speech therapy clinics, utilized as a measure of neurological capacity. A study^(24)^ observed a progressive reduction in the number of syllables per second, depending on the degree of effort required for the production of the syllable. In other words, the syllable /pa/, as it is a labial phoneme and utilizes only the orbicularis muscle, exhibited a greater number of syllables per second than the syllable /ka/, where the degree of effort increases due to the greater number of muscles involved and because it is a stop velar consonant. The present study revealed that the syllables /pa/ and /ta/ did not undergo significant changes in their values when comparing the pre-and post-moments. A study^(13)^ conducted asserts that diadochokinesia must be evaluated even in the absence of neurological issues, as alterations in the extension and velocity of the vocal folds can reflect changes in the production rate, duration patterns, and transglottic airflow speed.

This study found significant differences with an increase in the number of repetitions of the syllables /ka/ (p=0.012), /a/ (p=0.005), and /pataka/ (p=0.009). Diadochokinesis, in addition to observing intact neuromotor conditions and central nervous system control, requires that morphological and behavioral aspects be intact for the test to be performed. With the diadochokinesis test, it is possible to observe that training promoted greater aerodynamic and diadochokinesis control, since it requires the balance of muscular forces to perform the test.

As part of the comprehensive vocal assessment, self-assessment plays a pivotal role in the vocal clinic, as it allows for a subjective evaluation of the participant's perception of their own voice^(27)^. In this study, it is assumed that the participants are future voice professionals who would benefit from improved vocal efficiency.

As for the vocal self-assessment performed in this study, it is possible to observe an increase in the median total and physical QOL scores (cut-off values 91.25 and 89.60, respectively)^(16)^. The increased scores observed in these results after inspiratory training suggest a significant improvement in the participants' perception of vocal quality. In the IDV-10, there was a decrease in the median score after training (cut-off value 7.5 points)^(1,6)^, indicating a significant reduction in the perception of vocal disadvantage, which suggests a positive impact of inspiratory training. The ESV protocol showed a decrease in the medians of the total score and the limitation and physical subscales when comparing pre- and post-training (cut-off values: 16, 11.55, and 6.5, respectively)^(18)^.

These findings related to vocal self-assessment, with values below the cut-off points after inspiratory training, suggest an improvement in vocal symptoms, which were more noticeable to participants before the training. The improvement observed in the vocal self-assessment instruments may be related to the physiological adjustments promoted by inspiratory training. Studies indicate that breathing exercises can positively influence vocal parameters such as subglottic pressure and airflow, contributing to more efficient vocal production^(3,10,11)^. In addition, inspiratory muscle training can increase the strength of the respiratory muscles^(10-12)^, resulting in greater control and stability during phonation^(1,10-12)^. In general, it was observed that the participants experienced an increase in voice quality of life, a decrease in vocal disadvantage, and a decrease in vocal symptoms after performing inspiratory training with Respiron® Classic for 28 consecutive days.

No other studies comparing vocal self-assessment before and after inspiratory training with the Respiron® Classic respiratory incentive device were found in the literature.

In consideration of the aforementioned findings, alterations in acoustic measurements were observed, indicating an enhancement in the phonatory mechanism, an increase in aerodynamic measurements and diadochokinesis, and an improvement in vocal self-assessment. These changes were accompanied by an enhancement in voice quality of life, a reduction in vocal handicap, and a decrease in vocal signs and symptoms in women without vocal complaints who underwent inspiratory muscle training with Respiron® Classic for 28 days. It is important to mention that the effect size results in statistical differences, moderate to large, indicate that the intervention had a significant and relevant impact on the variable analyzed. This suggests that the difference between the groups is not only statistically significant, but also has sufficient practical magnitude to be considered important in real-world application.

Studies in the field of voice related to dysphonia have been published; however, there is a gap in relation to the parameters necessary to verify the effectiveness of vocal training with vocally healthy individuals, especially since such resources have also been used in professional voice training, mainly with a view to vocal conditioning for people who use their voice artistically.

The choice of Respiron® Classic, an inspiratory flow training device, allows for the use of a low-cost resource for respiratory training, but it does not allow for verification of the exact level of pressure exerted during its use. However, for each adjustment level, the manufacturers (NCS) estimate an approximate pressure value in cm H2O during training, as follows: 15 cm H2O to raise the three spheres at adjustment zero, 25 cm H2O at adjustment one, 30 cm H2O at adjustment two, and 40 cm H2O at adjustment three. Even so, this choice implies a methodological limitation due to the lack of calibratable control of inspiratory resistance.

Based on the assumption that different levels of effort generate significant gains in lung capacity and respiratory muscle strength, studies are needed to explore vocal differences for each level of effort, using calibratable Inspiratory Muscle Training devices such as Power Breathe® and Threshold®; in individuals with vocal disorders; voice professionals as a resource for vocal conditioning and the training necessary to gain and maintain vocal quality over time, such research will contribute to scientific progress in the field.

## CONCLUSION

Based on the results obtained, it can be concluded that inspiratory training with the Respiron® Classic Respiratory Exerciser and Incentivator ® Classic for 28 days can increase the maximum phonation times of the sustained vowel /i/, the fricatives /s/ and /z/, and the number count, decrease the acoustic measurement of shimmer and increase the measurement of HNR (Harmonic-to-Noise Ratio), increase in the number of syllable repetitions in the diadochokinesis test of /ka/, /a/ and /pataka/ and in the self-assessment increase in voice quality of life by the QVV protocol physical and total scores, and decrease in vocal disadvantage by the IDV-10 protocols and decrease in vocal signs and symptoms by the ESV scale, physical limitation and total scores.

Thus, it can be inferred that inspiratory training promoted greater phonatory efficiency and increased aerodynamic control. Therefore, inspiratory muscle training is encouraged for subjects without vocal complaints who wish to increase their phonatory efficiency.

## Appendix A Protocol for scheduling inspiratory training with Respiron® Classic for 28 consecutive days

Name: ______________________________________________________________________

**Table:**

**Orientation**
Each day of training, you must record the date of the exercise in the corresponding field. This procedure must be repeated for 28 consecutive days, according to the equipment user manual. After completion, you must fill in the fields corresponding to the series and the level of effort performed.

**Table:**

1st week	1st day	2nd day	3rd day	4th day	5th day	6th day	7th day
Date:							
2 sets of 30 repetitions	( ) yes	( ) yes	( ) yes	( ) yes	( ) yes	( ) yes	( ) yes
	( ) no	( ) no	( ) no	( ) no	( ) no	( ) no	( ) no
Weight:	( ) 0	( ) 0	( ) 0	( ) 0	( ) 0	( ) 0	( ) 0
	( ) 1	( ) 1	( ) 1	( ) 1	( ) 1	( ) 1	( ) 1
	( ) 2	( ) 2	( ) 2	( ) 2	( ) 2	( ) 2	( ) 2
	( ) 3	( ) 3	( ) 3	( ) 3	( ) 3	( ) 3	( ) 3

**Table:**

2nd week	1st day	2nd day	3rd day	4th day	5th day	6th day	7th day
Date:							
2 sets of 30 repetitions	( ) yes	( ) yes	( ) yes	( ) yes	( ) yes	( ) yes	( ) yes
	( ) no	( ) no	( ) no	( ) no	( ) no	( ) no	( ) no
Weight:	( ) 0	( ) 0	( ) 0	( ) 0	( ) 0	( ) 0	( ) 0
	( ) 1	( ) 1	( ) 1	( ) 1	( ) 1	( ) 1	( ) 1
	( ) 2	( ) 2	( ) 2	( ) 2	( ) 2	( ) 2	( ) 2
	( ) 3	( ) 3	( ) 3	( ) 3	( ) 3	( ) 3	( ) 3

**Table:**

3rd week	1st day	2nd day	3rd day	4th day	5th day	6th day	7th day
Date:							
2 sets of 30 repetitions	( ) yes	( ) yes	( ) yes	( ) yes	( ) yes	( ) yes	( ) yes
	( ) no	( ) no	( ) no	( ) no	( ) no	( ) no	( ) no
Weight:	( ) 0	( ) 0	( ) 0	( ) 0	( ) 0	( ) 0	( ) 0
	( ) 1	( ) 1	( ) 1	( ) 1	( ) 1	( ) 1	( ) 1
	( ) 2	( ) 2	( ) 2	( ) 2	( ) 2	( ) 2	( ) 2
	( ) 3	( ) 3	( ) 3	( ) 3	( ) 3	( ) 3	( ) 3

**Table:**

4th week	1st day	2nd day	3rd day	4th day	5th day	6th day	7th day
Date:							
2 sets of 30 repetitions	( ) yes	( ) yes	( ) yes	( ) yes	( ) yes	( ) yes	( ) yes
	( ) no	( ) no	( ) no	( ) no	( ) no	( ) no	( ) no
Weight:	( ) 0	( ) 0	( ) 0	( ) 0	( ) 0	( ) 0	( ) 0
	( ) 1	( ) 1	( ) 1	( ) 1	( ) 1	( ) 1	( ) 1
	( ) 2	( ) 2	( ) 2	( ) 2	( ) 2	( ) 2	( ) 2
	( ) 3	( ) 3	( ) 3	( ) 3	( ) 3	( ) 3	( ) 3

Source: The authors (2025)

## References

[1] Behlau M (2005). Voz: o livro do especialista I..

[2] Leão RLDS, Gomes ADOC, Queiroz MRG, Lucena JA (2022). Voice therapy with a respiratory approach in older people: an integrative literature review. Rev CEFAC.

[3] Machado ÉC, Frigo LF, Bresolin FA, Lima JPDM, Cielob CA (2020). Immediate effects of cervical stimulation and diaphragmatic release on vocal production. Fisioter Mov.

[4] Sloboditcov LDS (2017). Ação da tarefa de força expiratória na atividade elétrica dos músculos extrínsecos da laringe em adultos saudáveis.

[5] Cruz MDSL (2013). Análise da resistência do incentivador respiratório em indivíduos jovens. Fisioter Bras.

[6] Rosa NN, Escorcio R (2020). Análise da eficácia de incentivador respiratório a fluxo sobre a força muscular respiratória, resistência e tolerância ao exercício em pacientes com doença pulmonar obstrutiva crônica. Rev Fac Ciênc Méd Sorocaba..

[7] Rodrigues AAW (2013). Respiron® Exercitador e incentivador respiratório: manual de uso..

[8] Sapienza C, Troche M, Pitts T, Davenport P (2011). Respiratory strength training: concept and intervention outcomes. Semin Speech Lang.

[9] Alves TC, de Souza AMT, do Espirito Santo ECB, Godinho P, Chicayban L (2023). Treinamento muscular inspiratório com incentivador a fluxo em idosos institucionalizados: ensaio clínico randomizado duplo-cego. Biológicas & Saúde.

[10] Lopes BP, Korn GP, Nunes FB, Gama ACC (2023). Immediate effects of the incentive spirometer in women with healthy voice. CoDAS.

[11] Beraldo AT, Batistella J, Martins PDN, Dassie-Leite AP, Pereira EC (2024). Immediate effect of inspiratory exercise with exerciser and respiratory encourager in women without vocal complaints. CoDAS.

[12] Bordignon F, Cardoso MCAF (2016). Clinical parameters of speech therapy respiratory function from the use of inspiratory encourager. Distúrbios Comum..

[13] Oliveira MD, Santos CLS, Oliveira CFD, Ribas DIR (2013). Effects of expansive technical and incentive spirometry in respiratory muscle strength in institutionalized elderly. Fisioter Mov.

[14] Siqueira LTD, Silverio KCA, Brasolotto AG, Guirro RRJ, Carneiro CG, Behlau M (2017). Effects of laryngeal manual therapy (LMT) and transcutaneous electrical nerve stimulation (TENS) in vocal folds diadochokinesis of dysphonic women: a randomized clinical trial. CoDAS.

[15] Ghirardi ACA, Ferreira LP, Giannini SPP, Latorre MRDO (2013). Screening Index for Voice Disorder (SIVD): development and Validation. J Voice.

[16] Hogikyan ND, Sethuraman G (1999). Validation of an instrument to measure voice-related quality of life (V-RQOL). J Voice.

[17] Gasparini G, Behlau M (2009). Quality of life: validation of the Brazilian version of the Voice-Related Quality of Life (V-RQOL) measure. J Voice.

[18] Rosen CA, Lee AS, Osborne J, Zullo T, Murry T (2004). Development and validation of the Voice, Handicap Index-10. Laryngoscope.

[19] Costa T, Oliveira G, Behlau M (2013). Validation of the Voice Handicap Index – 10 (VHI-10) to the Brazilian Portuguese. CoDAS.

[20] Moreti F, Zambon F, Oliveira G, Behlau M (2014). Cross-cultural adaptation, validation, and cutoff values of the Brazilian version of the voice symptom scale – VoiSS. J Voice.

[21] Yamasaki R, Madazio G, Leão SH, Padovani M, Azevedo R, Behlau M (2017). Auditory-perceptual evaluation of normal and dysphonic voices using the voice deviation scale. J Voice.

[22] Lopes LW, Sousa ESS, Silva ACF, Silva IM, Paiva MAA, Vieira VJD (2019). Cepstral measures in the assessment of severity of voice disorders. CoDAS.

[23] Englert M, Lima L, Constantini AC, Latoszek BBV, Maryn Y, Behlau M (2019). Acoustic Voice Quality Index-AVQI for Brazilian Portuguese speakers: analysis of different speech material. CoDAS.

[24] Englert M, Lima L, Behlau M (2020). Acoustic voice quality index and acoustic breathiness index: analysis with different speech material in the Brazilian Portuguese. J Voice.

[25] Padovani M, Gielow I, Behlau M (2009). Phonarticulatory diadochokinesis in young and elderly individuals. Arq Neuropsiquiatr.

[26] Behlau M, Almeida AA, Amorim G, Balata P, Bastos S, Cassol M (2022). Reducing the GAP between science and clinic: lessons from academia and professional practice - part A: perceptual-auditory judgment of vocal quality, acoustic vocal signal analysis and voice self-assessment. CoDAS.

[27] Moura LMGMO, Silva POC, Santana ÉR, Batista DJ, Duarte JMT, Ribeiro VV (2023). Measurements of the effect of interventions on heathly voices: a scope review. Audiol Commun Res.

[28] Finger LS, Cielo CA, Schwarz K (2009). Acoustic vocal measures in women without voice complaints and with normal larynxes. Braz J Otorhinolaryngol.

[29] Wertzner HF, Schreiber S, Amaro L (2005). Analysis of fundamental frequency, jitter, shimmer and vocal intensity in children with phonological disorders. Braz J Otorhinolaryngol.

[30] Lopes J, Freitas S, Sousa R, Matos J, Abreu F, Ferreira A (2008). A medida HNR: sua relevância na análise acústica da voz e sua estimação precisa..

