# Supplementary material for: Effects of inspiratory muscle training for 28 days on the voice of women without vocal complaints
Source: Codas. 2026 Mar 2;38(1):e20250109. doi: 10.1590/2317-1782/e20250109en (PMC12978322; doi:10.1590/2317-1782/e20250109en)
Supplement: Translation

# Translation: Efeitos do treinamento muscular inspiratório por 28 dias na voz de mulheres sem queixas vocais

**DOI:** 10.1590/2317-1782/e20250109pt

**Authors:** Julia Batistella, Alessandra Thais Beraldo, Perla do Nascimento Martins, Ana Paula Dassie-Leite, Eliane Cristina Pereira

**Affiliations:** 1 Departamento de Fonoaudiologia, Universidade Estadual do Centro-Oeste – UNICENTRO, Irati (PR), Brasil.

**Keywords:** Exercícios Respiratórios, Voz, Qualidade Vocal, Respiração, Treinamento de Voz

## Abstract

**Objetivo:**

Analisar os efeitos vocais do treinamento muscular inspiratório por 28 dias em mulheres sem queixas vocais.

**Método:**

Participaram do estudo 22 mulheres sem queixas vocais, que realizaram treinamento inspiratório com Exercitador e Incentivador Respiratório Respiron® Classic. O treinamento consistiu em 2 séries por dia, de 30 repetições, durante 28 dias consecutivos. Amostras vocais foram coletadas pré e pós treinamento e comparadas quanto a: julgamento perceptivo-auditivo (JPA), análise acústica, medidas aerodinâmicas, diadococinesia e autoavaliação vocal. Os dados foram analisados estatisticamente por meio de testes dependentes.

**Resultados:**

Não foram encontradas diferenças no JPA da vogal sustentada /a/ e da contagem de números. Foram encontradas diferenças na análise acústica, com diminuição da mediana de Shimmer % e aumento de HNR. Houve aumento das médias dos tempos máximos de fonação da vogal /i/, das fricativas /s/ e /z/ e da contagem de números. Houve, ainda, aumento das médias do número de repetição de sílabas na diadococinesia das sílabas /ka/ e /pataka/ e da vogal /a/. Aumento das médias do protocolo de autoavaliação QVV físico e QVV total, e diminuição do IDV-10; ESV limitação, ESV físico e ESV total.

**Conclusão:**

Pode-se inferir que o treinamento inspiratório promoveu maior eficiência fonatória e aumento do controle aerodinâmico, propiciando uma melhora na qualidade vocal. Assim sendo, incentiva-se o treinamento muscular inspiratório para sujeitos sem queixas vocais que desejem obter aumento de sua eficiência fonatória.

## INTRODUÇÃO

A produção da voz depende de pressão aérea adequada, a qual é fundamental para ocorrer a aproximação e vibração das pregas vocais de maneira eficiente^(1-3)^. Nesse sentido, exercitadores e incentivadores respiratórios têm sido utilizados na clínica vocal com o objetivo de melhorar a eficiência fonatória, isto é, a coordenação entre os subsistemas respiratório, glótico e ressonantal, que ocasiona uma produção de voz com mínimo esforço e máximo aproveitamento da energia respiratória^1-,4^, embora haja escassez de estudos na literatura que comprovem seus efeitos.

Incentivadores respiratórios são frequentemente utilizados pela área da fisioterapia com o intuito de se obter força muscular, tanto dos músculos inspiratórios quanto expiratórios. Atualmente, o uso desses dispositivos é comum em recuperação pós-cirurgias torácicas, além de serem indicados para o aumento da capacidade oxidativa, diminuição da fadiga e melhora no desempenho do sistema neuromuscular^(4-6)^.

O Exercitador e Incentivador Respiratório Respiron® *Classic* é um dispositivo que estimula a respiração a partir de esforço muscular. Ele incentiva uma inspiração profunda, com expansão máxima dos pulmões e da atividade muscular, principalmente do diafragma e dos demais músculos intercostais responsáveis pelo movimento respiratório. Por tratarem-se de músculos, estes podem ser treinados para promover o aumento da força e da resistência à fadiga^(7,8)^.

O Exercitador e Incentivador Respiratório Respiron® Classic é de fácil e indolor utilização, por meio de um conjunto de pesos para a musculatura inspiratória. Ainda possui estímulo visual (elevação das esferas) que se relaciona ao nível de dificuldade do exercício realizado. Tal modelo propõe exercícios que exigem esforço de nível médio, sendo o ideal para o início de exercícios respiratórios por pessoas sedentárias, obesas e idosas. Como benefícios do uso regular, tem-se diminuição do cansaço e da falta de ar, aumento da disposição na realização de atividades físicas, ganho de qualidade de vida e performance física^(7)^. Há grande possibilidade de evolução dentro de um período de três meses^(9)^, mas o início da evolução pode ser observado a partir da segunda semana de uso^(8)^.

Dois estudos investigaram o efeito imediato do exercício inspiratório com Respiron® Classic na voz de mulheres sem queixas vocais. Um deles, com 22 mulheres vocalmente saudáveis, identificou que o equipamento é seguro e seu efeito imediato promoveu redução nas medidas acústicas de aperiodicidade a curto prazo, relacionada à frequência e intensidade e aumento do volume expiratório máximo^(10)^. O segundo, realizado com 25 mulheres sem queixas vocais, concluiu que há aumento do valor da medida aerodinâmica do TMF /s/. Esse resultado demonstra o aprimoramento do controle aerodinâmico como efeito imediato do exercício inspiratório^(11)^.

Uma pesquisa com o mesmo equipamento evidenciou que o uso por quatro semanas aumenta a capacidade expiratória e melhora a eficiência laríngea. Foi possível observar aumento do PFE (pico de fluxo expiratório) e do VFE (volume expiratório forçado), no entanto sem atingir os valores normais esperados para adultos. Além disso, os valores do TMF da vogal /a/ e das fricativas /s/ e /z/ aumentaram^(12)^.

Outro estudo avaliou a Pressão Inspiratória máxima (PImáx), Pressão Expiratória máxima (PEmáx) e o pico de fluxo expiratório em 28 idosos institucionalizados, que realizaram o treinamento por meio de técnicas manuais expansivas e treinamento inspiratório, com incentivador respiratório Respiron® Classic. O treinamento foi realizado 3 vezes na semana, durante 6 semanas consecutivas, por 15 minutos, sendo um minuto de descanso a cada 4 minutos de treinamento. Na referida pesquisa, o Respiron® Classic utilizado isoladamente não modificou a força muscular respiratória em idosos, no entanto, ao ser associado com outras técnicas, ele contribuiu para o aumento da força muscular respiratória^(13)^.

O treinamento inspiratório por meio de exercitadores e incentivadores respiratórios é difundido atualmente na fonoaudiologia e utilizado frequentemente na prática clínica fonoaudiológica. Devido à escassez de literatura que elucide seus efeitos nas avaliações multidimensionais da voz, torna-se necessária a realização de estudos que busquem evidências sobre sua aplicabilidade e seus benefícios na clínica vocal.

Acredita-se que o treinamento respiratório visando a uma melhor eficiência fonatória seja de grande valia tanto para pessoas com distúrbios de voz quanto para pessoas vocalmente saudáveis, mas que desejam melhorar seu desempenho vocal no que se refere à qualidade, ressonância, intensidade/projeção e coordenação pneumofonoarticulatória. Isso porque, infere-se que a melhora da pressão subglótica contribui para a melhora da coaptação glótica, e consequentemente na qualidade da voz, aspecto que pode melhorar a diadococinesia, a qual é muito utilizada para avaliar o controle neuromotor das pregas vocais^(14)^.

Diante do exposto, o presente estudo teve o objetivo de comparar o julgamento perceptivo-auditivo, medidas acústicas, medidas aerodinâmicas, diadococinesia e a autoavaliação vocal pré e pós treinamento muscular com Exercitador e Incentivador Respiratório Respiron® Classic, por 28 dias na voz de mulheres sem queixas vocais.

## MÉTODO

Trata-se de um estudo de intervenção pré e pós-teste.

A pesquisa foi aprovada pelo Comitê de Ética em Pesquisa com seres humanos sob número 5.594.862. Todos os participantes assinaram o Termo de Consentimento Livre e Esclarecido (TCLE).

A coleta de dados ocorreu entre os meses de novembro de 2023 e março de 2024. A amostra foi composta por 22 mulheres, com média de idade de 20,90 anos (+ 3,47), sem queixas vocais, estudantes universitárias de cursos da área da saúde. A coleta de dados de avaliação vocal foi realizada em laboratório de voz de uma Universidade.

No dia da primeira coleta, as mulheres foram orientadas sobre como realizar o treinamento muscular inspiratório com Exercitador e Incentivador Respiratório Respiron® Classic, por 28 dias, a fim de compreender o funcionamento do treinamento, este deveria ser marcado em um protocolo próprio, conforme o Apêndice A.

As participantes realizaram uma série juntamente com a pesquisadora e puderam tirar suas dúvidas sobre a utilização e execução. As participantes foram orientadas a realizar o treinamento inspiratório em casa por 28 dias consecutivos. Nesse período, a pesquisadora entrou em contato com as participantes semanalmente para conferir se a realização estava sendo executada corretamente e esclarecer eventuais dúvidas sobre o processo.

Os critérios de inclusão foram: sexo feminino, estudantes dos cursos de graduação, com idade acima de 18 anos.

Os critérios de exclusão foram presença de queixas vocais, mensuradas por meio protocolo de Índice de Triagem para Distúrbio da Voz (ITDV)^(15)^, o qual é um instrumento validado para a triagem vocal e consiste em um questionário de 12 questões, sendo que, cada questão respondida com “frequentemente” e “sempre” soma um ponto. Ao atingir 5 pontos ou mais, tem-se indicativo de alteração vocal. Foram excluídos da pesquisa também, indivíduos tabagistas, que autorreferiram alterações de via aérea crônicas ou no dia da coleta; ter contraído COVID-19 até um mês antes da coleta ou complicações durante ou após a contaminação pela COVID-19; que realizam ou realizaram tratamento vocal ou cirurgia na laringe; que autorrelataram doenças hormonais, neurológicas, psiquiátricas, pulmonares, perdas auditivas e que não participaram de alguma etapa da pesquisa.

### Execução do treinamento muscular inspiratório

As participantes realizaram o treinamento muscular inspiratório, sendo 2 séries por dia, cada uma delas com 30 repetições, com o Exercitador e Incentivador Respiratório Respiron® Modelo Classic nível médio, por 28 dias consecutivos.

Todas as participantes receberam a orientação para realizar as séries sentadas, inspirando fortemente até elevar as três esferas do Respiron® Classic e tentando sustentar por até três segundos, com cuidado para não elevar os ombros e contrair os músculos na região do pescoço.

Os exercícios foram iniciados no nível 0 com todas as participantes, que corresponde ao nível de esforço mínimo. Além disso, as participantes registraram seus progressos em um protocolo próprio, onde anotaram a data do treinamento, quantas vezes realizaram durante o dia e o grau de esforço (de zero a três) realizado no dia respectivo (Apêndice A).

A presente pesquisa não determinou um nível de esforço específico para as participantes realizarem a execução do treinamento ao longo das semanas. Sendo assim, foi orientado que as participantes aumentassem o grau de esforço (nível) quando todas as esferas do equipamento estivessem sendo elevadas sem esforços ao final das 30 repetições.

Para a análise das amostras vocais, levou-se em conta as participantes que realizaram o treinamento muscular inspiratório todos os dias, admitiu-se a falha uma única vez na semana. A pesquisadora manteve contato com as participantes, com a finalidade de conversar uma vez por semana sobre como estava a realização do treinamento e responder possíveis dúvidas.

### Coletas dos dados

A coleta dos dados e das amostras vocais ocorreu da seguinte maneira:

1. Preenchimento do questionário acerca dos dados de identificação, queixas vocais, histórico de doenças, hábitos nocivos e saudáveis e do protocolo de triagem vocal ITDV^(15)^;
2. Preenchimento dos protocolos de autoavaliação vocal: Qualidade de Vida em Voz^(16)^, Índice de Desvantagem Vocal 10 (IDV 10)^(17)^ e Escala de Sintomas Vocais (ESV)^(18)^;
3. Gravação da voz em laboratório acusticamente tratado, LABORVOZ, por meio do Programa de gravação de voz Audacity, em Computador Desktop, CPU AMD Athlon(tm) 64 X2 Dual Core Processor 5000+, Memória 4GB, SSD 240GB, sistema operacional Windows 7 Pro - 32 bits, utilizando-se microfone unidirecional da marca Shure modelo SM58, acoplado a interface de áudio M Audio Fast Track, posicionado frontalmente à boca, em distância de 5 centímetros. As gravações foram realizadas com taxa de amostragem de 44100 Hz, 16 bits de resolução, mono canal e em formato *wave*. As amostras gravadas foram:

- Vogal sustentada /a/;
- Contagem de números de 1 a 20;
- Tempo máximo de fonação das vogais /a/, /i/ /u/, fonemas /s/ e /z/ e contagem de números;
- Diadococinesia (contagem do número de repetições de sílabas) com /pa/, /ta/ e /ka/, palavra /pataka/ e fonema /a/.

Após os 28 dias, as vozes foram gravadas novamente, seguindo as etapas 2, 3 e 4 para a coleta do pós-intervenção.

Embora a captação inicial tenha sido de 24 participantes, durante a realização da pesquisa duas delas foram excluídas. Uma delas contraiu o vírus da COVID-19 durante o treinamento. A segunda não havia comentado no momento da anamnese que apresentava refluxo gastroesofágico e, no início da realização do treinamento, o refluxo se intensificou, o que foi relatado à pesquisadora, que optou por orientar a descontinuidade da prática.

### Julgamento perceptivo-auditivo

Uma juíza, fonoaudióloga especialista em voz, com experiência em análise vocal de 10 anos, recebeu uma pasta com as vozes aos pares, sem que soubesse qual era o momento pré e qual era o momento pós. Ela julgou as análises pela Escala de Desvio Vocal- EDV^(19)^, utilizando as amostras vocais da vogal /a/ sustentada e contagem de números de 1 a 10, de acordo com o grau geral do desvio vocal em escala visual analógica de 100 mm.

Foi realizada a análise de concordância interna da juíza, pelo teste estatístico Coeficiente de Correlação Intraclasse (CCI), repetindo-se 20% da amostra, de modo aleatório, referente à Escala de Desvio Vocal. Após análise pelo CCI, verificou-se que os pares tiveram concordância entre si, todos com respostas acima de 0,9, o que determina excelente concordância interna.

### Análise acústica da voz

Para a análise acústica foi utilizado o software PRAAT, sendo extraídas as medidas acústicas. Foram analisados os 3 segundos mediais da emissão da vogal /a/ sustentada, o que exclui instabilidades iniciais e finais da emissão. Com essa amostra foram obtidas as medidas de: Frequência fundamental; medidas de perturbação (*jitter* e *shimmer*), medida de ruído *Harmonic-to-Noise Ratio* (HNR). A medida cepstral *Cepstral Peak Prominence-Smoothed* (CPPS) foi extraída da vogal sustentada /a/ e da contagem de números^(20)^. A medida multiparamétrica *Acoustic Voice Quality Index* (AVQI) também foi obtida, considerando-se as amostras de vogal e a contagem de números (1 a 11)^(21)^.

Foi extraída, ainda, a medida *Acoustic Breathiness Index* (ABI), analisando em conjunto vogal e contagem, como no AVQI^(22)^.

### Medidas aerodinâmicas

Para a análise das medidas aerodinâmicas foram coletadas as medidas em segundos do Tempo Máximo de Fonação das vogais sustentadas /a/, /i/, /u/ e das fricativas surdas e sonoras /s/ e /z/, para posterior obtenção de sua relação^(1)^. A coleta de cada amostra foi realizada uma única vez e a extração do tempo foi feita programa Audacity.

### Diadococinesia

A análise da amostra vocal da diadococinesia foi realizada considerando o segundo medial da repetição de sílabas, contando o número de repetições em um segundo^(23)^.

### Autoavaliação vocal

Para a realização da autoavaliação vocal foram utilizados os protocolos:

-Qualidade de vida em voz (QVV)^(16,24)^: A pontuação dos domínios varia de 0 a 100, sendo que 0 indica baixa qualidade de vida relacionada à voz e 100 indica excelente qualidade de vida relacionada à voz. Os valores de corte estabelecidos para o QVV são de 91,25 para o Domínio Total, 89,60 para o Domínio Físico e 90,64 para o Domínio Socioemocional^(16)^.

-Índice de Desvantagem Vocal-10 (IDV-10)^(17,25)^: O escore total é calculado por meio da somatória simples das respostas que variam de 0 a 40 pontos, sendo que 0 indica nenhuma desvantagem vocal e 40 a desvantagem máxima. O valor de corte estabelecido para o IDV-10 é de 7,5 pontos^(17)^.

-Escala de Sinais e Sintomas Vocais (ESV)^(18)^: Os valores de corte estabelecidos para o ESV são de 16 para do domínio Total; 11,55 para a subescala limitação, 1,5 para a subescala emocional e 6,5 para a subescala físico^(18)^.

### Análise estatística

As variáveis dependentes (pré e pós treinamento) do estudo são os dados do julgamento perceptivo-auditivo, da análise acústica, das medidas aerodinâmicas e da diadococinesia. A variável independente, como se trata de estudo de intervenção, é o tempo de 28 dias utilizado para o treinamento, ou seja, o tempo entre os momentos da primeira e da segunda avaliação vocal

Os resultados foram comparados analisando-se as amostras dos momentos pré e pós treinamento. Anteriormente à análise inferencial dos resultados, foi realizado teste de normalidade de Shapiro-Wilk para análise de normalidade da distribuição dos dados. Dessa forma, foram utilizados os testes de Wilcoxon para a comparação de variáveis com distribuição não normal e T de Student pareado para a comparação de variáveis com distribuição normal. Nos casos de diferenças estatisticamente significantes, foi utilizado o teste d de Cohen para analisar o tamanho do efeito pós-T de Student Pareado e cálculo de (r) para analisar o tamanho de efeito pós-Wilcoxon.

O cálculo do tamanho da amostra foi estabelecido por meio de cálculo amostral pelo teste para Diferença Duas Proporções, a partir de um estudo semelhante que analisou o efeito do incentivador respiratório Shaker Plus antes e após três minutos de exercício. Os parâmetros fixos adotados para o teste foram α de 5%, β de 20% e K de 80%.

O tamanho da amostra mínimo calculado foi de 20 participantes nos grupos pré e no grupo pós treinamento.

## RESULTADOS

Participaram do estudo 22 mulheres com média de idade de 20,90 anos (+ 3,47). Todas as participantes eram estudantes universitárias e as amostras vocais foram constituídas por mulheres sem queixas vocais, as quais foram determinadas pelo protocolo de triagem vocal ITDV com resultado inferior a 5^(15)^.

Na Tabela 1 é possível visualizar a análise descritiva dos graus de esforço que cada mulher realizou o treinamento muscular inspiratório pré e pós 28 dias. Observa-se que a maior parte das participantes aumentou o nível de esforço ao longo do período de treinamento (Nível 0 – pré: 22/pós: 4; Nível 1 – pré: 0/pós: 7; Nível 2 – pré: 0/pós: 10; Nível 0 – pré: 0/pós: 1).

**Tabela 1:** Distribuição dos níveis de esforço realizados no treinamento muscular inspiratório pré e pós uso do exercitador e incentivador respiratório por 28 dias (n=22)

**MOMENTO**	**NÍVEL DE ESFORÇO**			
	Nível 0	Nível 1	Nível 2	Nível 3
Pré	22 (100%)	0 (0%)	0 (0%)	0 (0%)
Pós	4 (18,18%)	7 (31,81%)	10 (45,45%)	1 (4,45%)

Fonte: As autoras (2025)

Na Tabela 2 estão distribuídos os valores do julgamento perceptivo-auditivo do grau geral do desvio vocal da vogal /a/ sustentada e da contagem de números pré e pós realização do treinamento inspiratório por 28 dias. Não houve diferenças significativas nas comparações.

**Tabela 2:** Distribuição dos valores do julgamento perceptivo-auditivo do grau geral do desvio vocal da vogal /a/ e da contagem de números pré e pós realização do treinamento muscular inspiratório com exercitador e incentivador respiratório por 28 dias (n=22)

**GRAU GERAL DO DESVIO VOCAL DA VOGAL**							
		Média	Mediana	Mín.	Máx.	Desvio Padrão	P
**Vogal /a/**	Pré	42,36	42,00	24,00	55,00	7,88	0,495
	Pós	43,09	44,50	30,00	57,00	8,57	
**Contagem de Números**	Pré	29,04	26,00	10,00	49,00	12,09	0,380
	Pós	28,13	20,00	11,00	54,00	15,15	

Nota: Teste de Wilcoxon. Fonte: As autoras (2025)

Na Tabela 3 estão distribuídos os resultados referentes aos valores acústicos obtidos pré e pós a realização do exercício com incentivador respiratório por 28 dias. Houve diferença significativa entre os resultados de *shimmer (%)* (pré: 2,58/pós: 1,91) com tamanho de efeito negativo moderado a grande*,* e HNR (dB) (pré: 21,04/pós: 22,46), com tamanho de efeito positivo moderado a grande. Para as demais medidas não foram obtidas diferenças.

**Tabela 3:** Distribuição dos valores das medidas acústicas e dos protocolos de autoavaliação pré e pós treinamento muscular inspiratório com exercitador e incentivador respiratório por 28 dias (n=22)

**VALORES DAS MEDIDAS ACÚSTICAS**									
	Grupo	Média	Mediana	Mín	Máx	Desvio Padrão	p	Tamanho efeito	
**F0** 2	Pré	214,53	227,11	111,95	247,97	31,13	0,338		
	Pós	217,36	223,31	157,04	250,09	25,81			
**F0 números** ^2^	Pré	210,81	216,05	161,61	214,41	20,39	0,262		
	Pós	225,87	219,19	137,29	531,56	72,67			
***Jitter*** ^2^	Pré	0,414	0,407	0,189	250,09	25,81	0,485		
	Pós	0,430	0,427	0,252	0,993	0,183			
***Shimmer*** ^2^	Pré	2,58	2,483	1,129	5,721	0,98	^*^ 0,008	-0.56	
	Pós	1,915	1,693	0,572	5,094	0,99			
**HNR**1	Pré	21,04	21,30	16,060	28,567	2,84	*0,026	0,51	
	Pós	22,46	22,74	16,331	29,698	3,68			
**CPPS /a/** ^1^	Pré	14,75	14,48	12,29	17,87	1,56	0,479		
	Pós	14,90	15,02	11,39	17,48	1,69			
**CPPS números** ^1^	Pré	9,03	8,81	7,04	11,06	1,02	0,428		
	Pós	9,14	9,19	6,21	11,07	1,27			
**AVQI** ^1^	Pré	1,665	1,47	0,14	3,030	0,83	0,778		
	Pós	1,698	1,68	0,080	3,45	0,91			
**ABI** ^1^	Pré	2,946	2,78	1,700	5,080	0,84	0,866		
	Pós	2,921	2,835	1,340	4,220	0,76			
	**VALORES DOS PROTOCOLOS DE AUTOAVALIAÇÃO**								
	Grupo	Média	Mediana	Mín	Máx	Desvio Padrão	p	Tamanho efeito	
	**QUALIDADE DE VIDA EM VOZ**								
**Domínio Físico** ^2^	Pré	88,25	91,70	66,70	100	9,58	*0,00	0,88	
	Pós	93,17	95,80	79,20	100	7,21			
**Domínio Socioemocional** ^2^	Pré	96,88	100	87,50	100	4,61	0,10		
	Pós	98,027	100	93,80	100	2,95			
**Total** ^2^	Pré	91,81	93,75	80,00	100	6,46	*0,00	0,88	
	Pós	95,25	97,50	87,50	100	4,75			
	**ÍNDICE DE DESVANTAGEM VOCAL-10**								
	Pré	3,00	2,00	0,00	18,00	4,05	*0,005	-0,89	
	Pós	1,81	1,00	0,00	9,00	2,44			
	**ESCALA DE SINTOMAS VOCAIS**								
**Limitação** ^2^	Pré	8,04	8,00	0,00	21,00	5,60	*0,00	-0,86	
	Pós	6,04	6,00	0,00	16,00	4,78			
**Emocional** ^2^	Pré	0,63	0,00	0,00	6,00	1,49	0,108		
	Pós	0,45	0,00	0,00	6,00	1,40			
**Físico** ^2^	Pré	4,81	4,50	1,00	12,00	2,80	*0,007	-0,82	
	Pós	3,86	3,00	0,00	10,00	2,49			
**Total** ^2^	Pré	13,68	12	2,00	28,00	7,54	*0,000	-0,88	
	Pós	10,72	8,50	1,00	25,00	6,65			

* Valores significativos

Nota Medidas Acústicas: ^1^Teste t de student para variáveis dependentes e tamanho do efeito com d de Cohen

2 Teste de Wilcoxon e tamanho do efeito com r de efeito.

Fonte: As autoras (2025)

Também foi realizada a comparação da autoavaliação vocal com os protocolos Qualidade de Vida em Voz (QVV)^(16)^, Índice de Desvantagem Vocal-10 (IDV-10)^(17)^ e Escala de Sinais e Sintomas Vocais (ESV)^(18)^, pré e pós o treinamento inspiratório por 28 dias (Tabela 3). Observam-se diferenças significativas nos valores das medianas dos protocolos do QVV domínio físico (pré 91,70 e pós 95,80, valor de p:0,00 e tamanho de efeito 0,88) e total (pré 93,75 e pós 97,50, valor de p:0,00 tamanho de efeito 0,88), no protocolo IDV-10 (pré: 2 e pós 1, valor de p:0,005 tamanho de efeito -0,89) e na escala ESV limitação (pré: 8 e pós: 6, valor de p:0,00, tamanho de efeito -0.86) físico (pré: 4,5 e pós: 3, valor de p:0,007 tamanho de efeito -0,82) e total (pré: 12 e pós: 8,5, valor de p:0,00 tamanho de efeito -0,88). Todos os tamanhos do efeito (r) supracitados são considerados grandes, o que indica impactos substanciais sobre os desfechos analisados.

Na Tabela 4, estão distribuídos os valores das medidas aerodinâmicas referentes aos tempos máximos de fonação pré e pós. Observam-se diferenças significativas, com aumento dos valores no momento pós, nas medidas da vogal /i/ (pré: 13,86/pós: 15,90), das fricativas /s/ (pré: 13,36/pós: 16,04) e /z/ (pré: 13,54/pós: 16,18), e da contagem de números (pré: 17,68/pós: 19,50). Em todos os casos de diferenças, houve tamanho de efeito positivo moderado ou moderado a grande.

**Tabela 4:** Distribuição dos valores dos tempos máximos de fonação pré e pós treinamento muscular inspiratório com exercitador e incentivador respiratório por 28 dias (n=22)

		TEMPOS MÁXIMOS DE FONAÇÃO									
				Média	Mediana	Mín.	Máx.	Desvio Padrão	p		Tamanho Efeito
**TMF /a/** 2			Pré	14,22	13,00	9,00	29,00	4,81	0,159		
			Pós	15,09	14,50	8,00	25,00	3,97			
**TMF /i/** 1			Pré	13,86	14,00	8,00	23,00	3,31	^*^ 0,045		0,45
			Pós	15,90	15,00	9,00	28,00	4,50			
**TMF /u/** ^1^			Pré	13,63	14,00	8,00	22,00	3,23	0,129		
			Pós	14,90	14,00	10,00	23,00	3,13			
**TMF /s/** ^1^			Pré	13,36	14,50	5,00	24,00	4,42	*0,034		0,48
			Pós	16,04	16,00	6,00	27,00	5,68			
**TMF /z/** ^2^			Pré	13,54	13,00	6,00	27,00	4,76	*0,014		0,52
			Pós	16,18	15,00	9,00	34,00	5,59			
**TMF contagem de números** ^1^			Pré	17,68	17,50	11,00	29,00	4,49	*0,016		0,56
			Pós	19,5	19,50	11,00	37,00	5,73			
**Relação s/z** ^1^			Pré	1,01	0,96	0,60	1,63	0,28	0,79		
			Pós	0,995	1,05	0,62	1,35	0,22			

* Valores significativos

1 Teste t de student para variáveis dependentes e tamanho do efeito com d de Cohen

2 Teste de Wilcoxon e tamanho do efeito com r de efeito

Fonte: As autoras (2025)

Na Tabela 5 estão distribuídos os valores referentes ao número de repetições da prova de diadococinesia das sílabas /pa/, /ta/, /ka/ /pataka/ e da vogal /a/ pré e pós. Observam-se diferenças significativas, com aumento do número de sílabas produzidas no momento pós, nas medidas de /ka/ (pré: 5,90/pós: 6,68), /a/ (pré: 4,63/pós: 5,40) e /pataka/ (pré: 7,72/pós: 8,45). As diferenças estatísticas tiveram tamanho de efeito de moderado a grande.

**Tabela 5:** Distribuição dos valores da diadococinesia pré e pós o treinamento muscular inspiratório com exercitador e incentivador respiratório por 28 dias (n=22)

VALORES DA DIADOCOCINESIA								
		Média	Mediana	Mín.	Máx.	Desvio Padrão	p	Tamanho Efeito
**PA**	Pré	6,36	7,00	4,00	7,00	0,84	0,806	
	Pós	6,50	7,00	5,00	8,00	1,144		
**TA**	Pré	6,40	6,00	4,00	8,00	1,05	0,147	
	Pós	6,81	7,00	5,00	9,00	1,18		
**KA**	Pré	5,90	6,00	4,00	8,00	1,01	*0,012	0,58
	Pós	6,68	6,50	5,00	9,00	1,12		
**A**	Pré	4,63	5,00	3,00	6,00	0,78	*0,005	
	Pós	5,40	5,00	4,00	7,00	0,85		0,67
**PATAKA**	Pré	7,72	8,00	6,00	10,00	0,98	*0,009	0,65
	Pós	8,45	8,50	7,00	10,00	0,96		

* Valores significativos

Teste de Wilcoxon com r de efeito

Fonte: As autoras (2025)

## DISCUSSÃO

O treinamento inspiratório consiste em uma série de exercícios que estimulam a coordenação de grupos musculares necessários para a respiração. Compreende-se que estes músculos devem trabalhar em conjunto para que se possa fornecer a quantidade de ar necessária para desencadear a produção vocal de maneira eficiente. Sendo assim, o treinamento inspiratório apresenta relevância na prática clínica fonoaudiológica, pois influencia o fluxo de ar transglótico e a pressão subglótica, favorecendo a produção vocal e contribuindo para a melhora da qualidade vocal^(3,11)^.

As participantes do estudo iniciaram o treinamento respiratório no nível de esforço zero e não foi estabelecido um nível de esforço para o término do treinamento. Na Tabela 1, é possível observar que a maioria das participantes terminou o treinamento no nível de esforço dois e somente uma evoluiu para o último nível. Um estudo^(5)^ adaptou um manovacuômetro no exercitador e incentivador respiratório Respiron® Classic, com o objetivo de investigar a resistência gerada nos diferentes níveis de graduação do equipamento Respiron® Classic em indivíduos jovens, por meio da pressão inspiratória máxima. O estudo concluiu que a resistência gerada pelo equipamento é de leve a moderada, para os níveis 1, 2 e 3 e que as variações dos níveis de esforço refletem em diferenças significativas nas medidas finais da pressão inspiratória máxima.

Não foram observadas diferenças no julgamento perceptivo-auditivo do grau geral do desvio vocal pela Escala de Desvio Vocal (EDV), comparando-se os momentos pré e pós treinamento. Tal dado mostra que o efeito do uso do dispositivo pelo tempo empregado na pesquisa não foi capaz de promover modificações na qualidade vocal do ponto de vista perceptivo-auditivo, e devido também à natureza do dispositivo utilizado que não promove ajustes laríngeos diretamente. Uma vez que o treinamento não envolveu aspectos laríngeos, mas somente respiratório, a hipótese era a de que, de fato, não haveria modificações importantes no JPA, sobretudo por se tratar de pessoas sem queixas e sem alterações importantes na escala de desvio. Pesquisas envolvendo o treinamento respiratório de pessoas com distúrbios vocais poderiam ter resultados diferentes sob essa perspectiva, uma vez que as mudanças laríngeas seriam mais significativas, o que impactaria em mudanças de qualidade vocal mais facilmente percebidas do ponto de vista perceptivo-auditivo. Por isso, encoraja-se a realização de novos estudos com essa população.

Em estudo realizado sobre o efeito imediato do uso do exercitador e incentivador respiratório Respiron® Classic, também não foram observadas mudanças na qualidade vocal por meio do julgamento perceptivo-auditivo^(11)^. Não encontrar diferenças no julgamento perceptivo-auditivo e encontrar nas medidas acústicas é um resultado comumente encontrado nas pesquisas, uma vez que é realizada a tentativa de relacionar medidas acústicas com a qualidade vocal percebida auditivamente, no entanto, as alterações em parâmetros acústicos isolados não costumam refletir perfeitamente o que o clínico ouve^(26)^.

Outro estudo que investigou os efeitos imediatos do uso do Respiron® Classic, em mulheres vocalmente saudáveis^(10)^, mostrou que houve melhora da qualidade vocal em 36,4% das participantes após uso de incentivador inspiratório, enquanto 50,0% não obtiveram alterações perceptíveis. Os autores mencionam que por tratar-se de mulheres não disfônicas, as mudanças na qualidade vocal seriam mais difíceis de serem percebidas, devido à homeostase do sistema Fonatório, e sugerem novos estudos com sujeitos disfônicos.

A análise acústica é o procedimento mais utilizado na área da voz para o monitoramento pré e pós intervenção, fornecendo estimativas únicas em relação às mudanças no processo de produção vocal^(27)^. Vale destacar que as mulheres da presente pesquisa não apresentavam queixas vocais, e são esperados que os valores das medidas estejam dentro ou muito próximos dos padrões de normalidade na análise do pré uso do exercitador e incentivador respiratório. Mesmo assim, houve modificações nos parâmetros.

No presente estudo, foi possível observar que o treinamento promoveu mudanças significativas nas medidas de *shimmer* % e dB (p=0,008), com mediana de 2,483 no pré e de 1,693 no pós, observando uma diminuição no valor da medida e estando dentro do padrão de normalidade esperado para mulheres que é de 1,393 a 4,861^(28)^. O *shimmer* é uma medida de estabilidade fonatória e que se encontra alterada quando há redução da resistência glótica, lesões de massa nas pregas vocais e maior ruído à emissão^(29)^. Esse achado corrobora resultados de estudo^(10)^ em que houve redução significativa nos valores de *jitter* (p<0,001), *shimmer* (p=0,011) e PPQ (p=0,001), indicativo de melhora na estabilidade e regularidade da voz.

O HNR % (p=0,026), apresentou aumento da média, evidenciando aumento da proporção de harmônicos sobre o ruído, com média de 21,04 no pré e de 22,46 no pós, ambas estando dentro dos padrões de normalidade esperados para a medida, entre 12,64 a 26,51^(28)^. A medida de Proporção Harmônico-Ruído (HNR %) traduz a eficiência do processo de fonação, isto é, quanto maior foi a eficiência do fluxo de ar expelido pelos pulmões em energia de vibração de pregas vocais e, quanto mais íntegro (saudável) for o ciclo vibratório, maior será a relação HNR^(30)^. Tais dados divergem de outro estudo^(11)^, que não observou modificações acústicas após o uso do Respiron® Classic. No entanto, a referida pesquisa se baseou numa análise de efeito imediato e não prolongado por 28 dias, como fez o presente estudo.

Em estudo que comparou o efeito imediato do exercício inspiratório com incentivador e exercitador respiratório Respiron® Classic não encontrou mudanças significativas nas medidas acústicas^(11)^.

A presente pesquisa identificou que o uso do dispositivo Respiron® Classic auxiliou na diminuição das medidas do *shimmer* e aumento do HNR, as quais mesmo estando dentro do padrão de normalidade modificaram no pós intervenção, já que quanto menores as medidas de *shimmer* e maiores as medidas de HNR, mais adequadas se mostram as medidas acústicas. Sendo assim, percebe-se que o uso do exercitador e incentivador respiratório auxilia no aumento da eficiência fonatória, uma vez que ambas as medidas estão relacionadas com a quantidade de ar necessário para a fonação.

Alterações no padrão respiratório podem aumentar o potencial diafragmático e, consequentemente, aumentar a função pulmonar respiratória e a capacidade de armazenamento de ar. Estudos concluíram que o uso de incentivador respiratório causa efeito na dinâmica respiratória e propicia um fluxo aerodinâmico adequado, o que gera um maior controle vocal e maior tempo de emissão^(3,12)^. No presente estudo, foram encontradas diferenças significativas nos tempos máximos de fonação das vogais /i/, das fricativas /s/ e /z/ e da contagem de números.

Um estudo sobre o efeito imediato do uso do incentivador respiratório Respiron® Classic também observou aumento no valor do TMF do fonema /s/. Os autores sugeriram que o aumento dessa medida evidencia um melhor controle aerodinâmico e aumento da capacidade pulmonar^(11)^. Percebeu-se a vantagem do treinamento inspiratório para a modificações das medidas aerodinâmicas diante da execução de uma única realização. Resultados semelhantes foram encontrados em pesquisa realizada com cinco indivíduos vocalmente saudáveis^(12)^, onde foi observado aumento do TMF nas médias finais de produção das vogais e fricativas e que sugeriu que o uso de incentivadores respiratórios propicia aumento do controle aerodinâmico. A pesquisa também observou que a evolução obtida se manteve após cinco semanas sem o uso do incentivador. Esses achados corroboram estudo^(10)^ que mostrou melhora nas medidas aerodinâmicas, com aumento significativo do volume expiratório máximo (p<0,001), sugerindo melhora na capacidade respiratória.

A medida aerodinâmica do TMF está diretamente relacionada com o fluxo aerodinâmico da respiração e fonação e das forças mioelásticas da laringe^(11,12)^. Sendo assim, é possível concluir pela literatura existente e pelos resultados do presente estudo que o uso do exercitador e incentivador respiratório Respiron® Classic promove o aumento do controle aerodinâmico e das forças mioelásticas da laringe.

O teste de diadococinesia é utilizado frequentemente na clínica fonoaudiológica e é considerado um teste de capacidade neurológica. Estudo^(23)^ observou a redução progressiva de sílabas por segundo, conforme o grau de esforço para a produção da sílaba aumentava. Ou seja, na sílaba /pa/ por ser um fonema labial e utilizar somente o músculo orbicular, o número de sílabas por segundo foi maior que na sílaba /ka/ onde o grau de esforço aumenta pelo número de músculos envolvidos ser maior e por ser uma consoante velar oclusiva. Na presente pesquisa, foi possível observar que apenas as sílabas /pa/ e /ta/ não obtiveram mudanças significativas em seus valores, comparando-se os momentos pré e pós. Um estudo realizado^(14)^ afirma que a diadococinesia deve ser avaliada mesmo na ausência de problemas neurológicos, uma vez que mudanças na extensão e velocidade das pregas vocais podem refletir em modificações na taxa de produção, nos padrões de duração e na velocidade de fluxo de ar transglótico.

A presente pesquisa obteve diferenças significativas com aumento do número de repetições das sílabas /ka/ (p=0,012), /a/ (p=0,005) e /pataka/ (p=0,009). A diadococinesia, além de observar condições neuromotoras íntegras e controle do sistema nervoso central, necessita que aspectos morfológicos e comportamentais estejam íntegros para a realização do teste^(14)^. Com o teste de diadococinesia, é possível observar que o treinamento promoveu maior controle aerodinâmico e da diadococinesia, uma vez que necessita do equilíbrio das forças musculares para a realização da prova.

Como parte das medidas que compõem a avaliação vocal, a autoavaliação é importante na clínica vocal, uma vez que leva em consideração a percepção do participante sobre a sua própria voz^(27)^. Leva-se em conta, na presente pesquisa, que as participantes do estudo são futuras profissionais da voz e que podem se beneficiar de uma melhor eficiência fonatória.

Quanto à autoavaliação vocal realizada na presente pesquisa, é possível observar aumento na mediana dos escores total e físico do QVV (valores de corte 91,25 e 89,60, respectivamente)^(16)^. O aumento observado nesses resultados, após o treinamento inspiratório, sugere uma melhora significativa na percepção da qualidade vocal pelas participantes. No IDV-10 houve diminuição no valor da mediana, no momento pós treinamento (valor de corte 7,5 pontos)^(17)^, indicativo de uma redução significativa na percepção de desvantagem vocal, o que sugere impacto positivo do treinamento inspiratório. Protocolo ESV mostrou diminuição das medianas no escore total e subescalas limitação e físico, na comparação entre pré e pós treinamento (valores de corte: 16, 11,55 e 6,5 respectivamente)^(18)^.

Esses achados relacionados à autoavaliação vocal, com valores abaixo dos pontos de corte após o treinamento inspiratório, sugerem uma melhora da sintomatologia vocal, que no momento pré era mais percebida pelas participantes. A melhora observada nos instrumentos de autoavaliação vocal pode estar relacionada aos ajustes fisiológicos promovidos pelo treinamento inspiratório. Estudos indicam que exercícios respiratórios podem influenciar positivamente parâmetros vocais, como pressão subglótica e fluxo aéreo, contribuindo para uma produção vocal mais eficiente^(3,10,11)^. Além disso, o treinamento muscular inspiratório pode aumentar a força dos músculos respiratórios^(10-12)^, resultando em maior controle e estabilidade durante a fonação^(1,10-12)^. De modo geral, observou-se que as participantes obtiveram aumento da qualidade de vida em voz, diminuição da desvantagem vocal e diminuição dos sintomas vocais, após a realização do treinamento inspiratório com Respiron® Classic por 28 dias consecutivos.

Na literatura, não foram encontrados outros estudos que compararam a autoavaliação vocal antes e após o treinamento inspiratório com incentivador respiratório Respiron® Classic.

Diante do exposto evidenciam-se modificações das medidas acústicas, demonstrando melhora do mecanismo fonatório, aumento das medidas aerodinâmicas e da diadococinesia. Na autoavaliação vocal foi observada melhora da qualidade de vida em voz, diminuição da desvantagem vocal e dos sinais e sintomas vocais de mulheres sem queixas vocais que realizaram treinamento muscular inspiratório com Respiron® Classic por 28 dias. É importante mencionar que os resultados de tamanho do efeito nas diferenças estatísticas, moderados a grandes, indicam que a intervenção teve um impacto significativo e relevante sobre a variável analisada. Isso sugere que a diferença entre os grupos não é apenas estatisticamente significativa, mas também possui uma magnitude prática suficiente para ser considerada importante na aplicação real.

Estudos são publicados na área da voz relacionados às disfonias, no entanto, observa-se uma lacuna em relação aos parâmetros necessários para a verificação da efetividade do treinamento vocal com indivíduos vocalmente saudáveis^(27)^, sobretudo porque tais recursos também têm sido utilizados em voz profissional, pensando-se principalmente no condicionamento vocal de pessoas que utilizam a voz artisticamente.

A escolha pelo Respiron® Classic, dispositivo de treino do fluxo inspiratório, possibilita o uso de um recurso de baixo custo para o treinamento respiratório, porém ele não permite verificar o nível exato de pressão exercida durante sua realização. Porém, para cada nível de regulagem, os fabricantes (NCS) estimam um valor aproximado da pressão em cm H_2_O à realização, sendo: 15 cm H_2_O para se elevar as três esferas na regulagem zero, 25 cm H_2_O na regulagem um, 30 cm H_2_O na regulagem dois e 40 cm H_2_O na regulagem três. Mesmo assim, tal escolha implica em uma limitação metodológica pela falta de controle calibrável de resistência inspiratória.

Partindo-se da hipótese de que os diferentes níveis de esforços geram ganhos significativos na capacidade pulmonar e na força muscular respiratória^(5)^, tornam-se necessários estudos que explorem as diferenças vocais para cada nível de esforço, com dispositivos para Treinamento Muscular Inspiratório calibráveis como Power Breathe® e Threshold®, em indivíduos com distúrbios vocais, profissionais da voz como recurso para o condicionamento vocal e o treinamento necessário para o ganho e a manutenção da qualidade vocal ao longo do tempo, tais pesquisas contribuirão para a evolução científica na área.

## CONCLUSÃO

A partir dos resultados obtidos, é possível concluir que o treinamento inspiratório com Exercitador e Incentivador Respiratório Respiron® Classic, por 28 dias, é capaz de promover o aumento dos tempos máximos de fonação da vogal sustentada /i/, das fricativas /s/ e /z/ e da contagem de números, promover diminuição da medida acústica de *shimmer* e aumento da medida de HNR (*Harmonic-to-Noise Ratio*), aumento do número de repetições de sílabas na prova de diadococinesia de /ka/, /a/ e /pataka/ e na autoavaliação aumento da qualidade de vida em voz pelo protocolo QVV escores físico e total, e diminuição da desvantagem vocal, pelo protocolos IDV-10 e diminuição dos sinais e sintomas vocais pela escala ESV, escores limitação físico e total.

Desse modo, pode-se inferir que o treinamento inspiratório promoveu maior eficiência fonatória e aumento do controle aerodinâmico. Assim sendo, incentiva-se o treinamento muscular inspiratório para sujeitos sem queixas vocais que desejem obter aumento de sua eficiência fonatória.

## Apêndice A Protocolo de marcação da realização do treinamento inspiratório com Respiron® Classic, por 28 dias consecutivos

Nome: __________________________________________________________________

**Table:**

**Orientações**
A cada dia de treinamento você deverá registrar a data de realização do exercício no campo correspondente.
Esse procedimento deverá ser repetido ao longo de 28 dias consecutivos, conforme manual de uso do equipamento1. Após a conclusão você deverá preencher os campos correspondentes às séries e ao nível de esforço realizado.

**Table:**

1ª semana	1º dia	2º dia	3º dia	4º dia	5º dia	6º dia	7º dia
Data:							
2 séries de 30 repetições	( ) sim	( ) sim	( ) sim	( ) sim	( ) sim	( ) sim	( ) sim
	( ) não	( ) não	( ) não	( ) não	( ) não	( ) não	( ) não
Carga:	( ) 0	( ) 0	( ) 0	( ) 0	( ) 0	( ) 0	( ) 0
	( ) 1	( ) 1	( ) 1	( ) 1	( ) 1	( ) 1	( ) 1
	( ) 2	( ) 2	( ) 2	( ) 2	( ) 2	( ) 2	( ) 2
	( ) 3	( ) 3	( ) 3	( ) 3	( ) 3	( ) 3	( ) 3

**Table:**

2ª semana	1º dia	2º dia	3º dia	4º dia	5º dia	6º dia	7º dia
Data:							
2 séries de 30 repetições	( ) sim	( ) sim	( ) sim	( ) sim	( ) sim	( ) sim	( ) sim
	( ) não	( ) não	( ) não	( ) não	( ) não	( ) não	( ) não
Carga:	( ) 0	( ) 0	( ) 0	( ) 0	( ) 0	( ) 0	( ) 0
	( ) 1	( ) 1	( ) 1	( ) 1	( ) 1	( ) 1	( ) 1
	( ) 2	( ) 2	( ) 2	( ) 2	( ) 2	( ) 2	( ) 2
	( ) 3	( ) 3	( ) 3	( ) 3	( ) 3	( ) 3	( ) 3

**Table:**

3ª semana	1º dia	2º dia	3º dia	4º dia	5º dia	6º dia	7º dia
Data:							
2 séries de 30 repetições	( ) sim	( ) sim	( ) sim	( ) sim	( ) sim	( ) sim	( ) sim
	( ) não	( ) não	( ) não	( ) não	( ) não	( ) não	( ) não
Carga:	( ) 0	( ) 0	( ) 0	( ) 0	( ) 0	( ) 0	( ) 0
	( ) 1	( ) 1	( ) 1	( ) 1	( ) 1	( ) 1	( ) 1
	( ) 2	( ) 2	( ) 2	( ) 2	( ) 2	( ) 2	( ) 2
	( ) 3	( ) 3	( ) 3	( ) 3	( ) 3	( ) 3	( ) 3

**Table:**

4ª semana	1º dia	2º dia	3º dia	4º dia	5º dia	6º dia	7º dia
Data:							
2 séries de 30 repetições	( ) sim	( ) sim	( ) sim	( ) sim	( ) sim	( ) sim	( ) sim
	( ) não	( ) não	( ) não	( ) não	( ) não	( ) não	( ) não
Carga:	( ) 0	( ) 0	( ) 0	( ) 0	( ) 0	( ) 0	( ) 0
	( ) 1	( ) 1	( ) 1	( ) 1	( ) 1	( ) 1	( ) 1
	( ) 2	( ) 2	( ) 2	( ) 2	( ) 2	( ) 2	( ) 2
	( ) 3	( ) 3	( ) 3	( ) 3	( ) 3	( ) 3	( ) 3

Fonte: As autoras (2025)
